# Supplementary material for: Understanding and comparing the medical tourism cancer patient with the locally managed patient: A case control study
Source: PLoS One. 2022 Sep 21;17(9):e0273162. doi: 10.1371/journal.pone.0273162 (PMC9491543; doi:10.1371/journal.pone.0273162)
Supplement: S1 File — (PDF) [file pone.0273162.s001.pdf]

QUESTIONAIRE ON CANCER PATIENTS' CHOICE OF TREATMENT CENTER

***STUDY ID NO.*____________________________**
***HEALTH FACILITY NAME:* _______________**
***COUNTRY*________________________________**

	**Instructions:**<br>• Use **Biro Pen** and write clearly<br>• Please tick **all** the responses or fill in the answers where it applies.<br>• Legibly **print** written responses.<br>• Mark inside the check box (preferably a cross), Not the code.<br>• Do **NOT** Mark more than one response to an item unless instructed to do so.<br>• Any mistakes should be corrected with a single line through wrong entry. Include your initials and date on corrected answer.
	**Name of Interviewer** ______________________________<br><br>**Was Consent Obtained?** ☐Yes 1 ☐No 2 If no, obtain consent before continuing.
1	**1.1.** Date of form completion **1.2** Participant Enrollment No.<br>|__|__|__|__|__|__|__|__|__| |__|__|__| dd mmm yyyy
2	Time of Interview **2.1** Start _____ : _____ **2.2** Finish: _____ : _____<br>(24 hour clock)
	**PART B: SOCIO-DEMOGRAPHIC DATA**
3	**What is your date of Birth** |__|__|__|__|__|__|__|__| *Don't know- 99 99 9999* **dd mm yyyy**
4	**Gender** ☐ Male1 ☐ Female2
6	Which County are you **currently living in (over last 1 year)?** _________________
	**6.1** Where exactly is **Residence located?** ☐ Urban area-1 ☐ Rural area-2
7	What is your **Marital Status**?<br>☐ Never married 1 ☐ Currently married- 2<br>☐ Separated- 3 ☐ Divorced- 4<br>☐ Widowed 5 ☐ Living together-6<br>☐ Not Applicable-7 ☐ Declined to answer99

QUESTIONAIRE ON CANCER PATIENTS' CHOICE OF TREATMENT CENTER

STUDY ID NO.______________________________
HEALTH FACILITY NAME: ________________
COUNTRY_________________________________

8 What is your the **highest level of education**?

☐ No formal schooling - 1 ☐ Some primary school - 2
☐ Primary School completed - 3 ☐ Secondary School completed - 4
☐ High school completed- 5 ☐ College (middle level, certificate, diploma) completed- 6
☐ University completed- 7 ☐ Postgraduate degree- 8
☐ Declined to answer- 99

9 What best describes you **main work** (**Occupation)?**

☐ Government employee - 1 ☐ Non-governmental organization - 2
☐ Unemployed- 3 ☐ Self-employed- 4
☐ Retired-5 ☐ Not Applicable-6 ☐ Other. 7 **9.1** Specify ______________

10 How long have you been in this **line of work** (occupation)?

☐ Less than 1 month-1 ☐ 1-6 months-2 ☐ 7-12 months-3 ☐ over 1 year-4 ☐ NA-5

11 What is your **average Monthly Income in Kenya Shillings? _________________**

PART C –DISEASE PROFILE

12 **What form of cancer were you diagnosed with?**

☐ Non-Hodgkin's Lymphoma-1 ☐ Colon-2
☐ Hodgkin's Lymphoma-3 ☐ Oral cavity- 4
☐ Rectum-5 ☐ Cervix- 6
☐ Breast- 7 ☐ Prostate- 8
☐ Leukemia- 9 ☐ Uterus-10
☐ Brain- 11 ☐ Esophagus -12
☐ Bone marrow -13 ☐ Pancreas 14
☐ Liver- 15 ☐ Lung -16
☐ Kaposi's Sarcoma-17 ☐ Other- 18 **12.1** Specify _____________

12.2 If yes, how long ago were you diagnosed? |__|__|__|__|__|__| mm yyyy
Don't remember- 99 9999

13 Have you ever been diagnosed with **any other type chronic diseases other than cancer?**
□ Yes-1 □ No-2 (go to Q 23) □ Decline to answer 99 (go to Q 23)

14

	Chronic Disease	Tick Yes	21. Date of diagnosis (mm/yyyy) *Don't remember- 99 9999*
14.1	High blood pressure disease		
14.2	Diabetes or high blood suga		
14.3	Chronic lung disease (e.g. asthma, Bronchitis)		
14.4	Obese		
14.5	Hepatitis B disease		
14.6	HIV		
14.7	Heart Disease		

15 **Specify any other chronic disease you may have?** ___________

15.1 **If yes, how long ago were you told you have <u>this chronic disease</u>?** |__|__|__|__|__|__| mm yyyy
Don't remember- 99 9999

PART D: INFORMATION ON CANCER CARE

16 What is the **Cancer treatment** you underwent in the first cycle/round of treatment?
☐ Chemotherapy-1 ☐ Radiotherapy- 2
☐ Surgery-3 ☐ Bone marrow transplant- 4
☐ Brachytherapy – 5 ☐ Don't know-99
☐ Other – 7 **16.1** Specify______________________

17 Did you also need to undergo a **procedure** at the same time?
☐ PET Scan-1 ☐ Radiological tests-2
☐ Diagnostic (laboratory) tests-3 ☐Don't know-99
☐ Other – 4 **17.1** Specify______________________

18 Who or what institution paid for your cancer treatment?

☐ Self-funding -1 ☐ NHIF -2

QUESTIONAIRE ON CANCER PATIENTS' CHOICE OF TREATMENT CENTER

STUDY ID NO.______________________________
HEALTH FACILITY NAME: ________________
COUNTRY_________________________________

	☐ Private Insurance Company -3 ☐ Employer -4
	☐ Other -5 **18.1** Specify _____________ ☐ Decline to answer -99
**19**	**Were you accompanied by a care giver?** ☐ Yes-1 ☐ No-2 (go to Q 22)
**20**	If Yes who be covering cost of the care giver?<br>☐ Self-1<br>☐ Themselves -2<br>☐ NHIF-3<br>☐ Insurance Company-4<br>☐ Friends /Relatives-5<br>☐ Other-6 **20.1** Specify _____________
**21**	If Yes what is your relationship with the care giver you have?<br>☐Spouse- 1 ☐ Friend- 2<br>☐ Family Member- 3 ☐ Other – 4 **21.1** Specify_____________
	**E. TREATMENT CENTER – FOR ALL PARTICIPANTS**
**22**	**Which health facility referred you for cancer treatment to the center you went?**<br>☐ Government hospital-1<br>☐ Private hospital-2<br>☐ Private clinic-3<br>☐ Other -4 **22.1** Specify ________________<br>**22.2** Which County is your referring health facility ______________________
**23**	**Who helped you organize your medical care for cancer?**<br>**23.1** Your local health care provider ☐ Yes-1 ☐ No-2<br>**23.2** Directly with the facility you are receiving care from ☐Yes-1 ☐No-2<br>**23.3** Self ☐ Yes-1 ☐No-2<br>**23.4** Friends and Relatives ☐Yes-1 ☐ No-2<br>**23.5** A local agent ☐ Yes-1 ☐ No-2<br>**23.6** Overseas agent ☐ Yes-1 ☐ No-2 ☐ NA-3

QUESTIONAIRE ON CANCER PATIENTS' CHOICE OF TREATMENT CENTER<br><br>***STUDY ID NO.______________________________***<br>***HEALTH FACILITY NAME: ________________***<br>***COUNTRY_________________________________***	
**24**	What is the **cost** of the treatment or procedure you received?<br>(In USD for treatment provided abroad and in Kshs for treatment provided in Kenya)<br>**24.1** Treatment _______________<br>**24.2** Procedure _______________<br>**24.3** Accommodation ______________<br>**24.4** Travel _______________
	**PART F : PATIENT RELATED INFLUENCING FACTORS**
**25**	What factors made you decide to choose the **HEALTH FACILITY** in which you received cancer treatment?<br><br>**25.1** Lack of adequate cancer treatment services where you were before? ☐Yes-1 ☐ No-2<br>**25.2** Amount of time you may have had to wait before treatment? ☐Yes-1 ☐ No-2<br>**25.3** If yes, please indicate how long you would have waited (in months) ___________<br><br>**25.4** Better quality of care ☐ Yes-1 ☐ No-2<br>**25.5** Friends/Relatives ☐ Yes-1 ☐ No-2<br>**25.6** Information from other patients who received treatment at location ☐Yes-1 ☐ No-2<br>**25.7** Advice from your local health care provider ☐ Yes-1 ☐ No-2<br>**25.8** Following a Medical camp in Kenya ☐ Yes-1 ☐ No-2<br>**25.9** Media Information sources. ☐ Yes-1 ☐ No-2<br>**25.10** If yes, what media source influenced your decision?<br>☐ Internet-1 ☐Radio-2 ☐ TV-3 ☐ Newspaper -4 ☐ Social media -5<br>☐ Other-6 **25.11** Specify_________________<br>**25.12** Cost effectiveness of treatment ☐ Yes-1 ☐No-2 ☐NA-3
**26**	**What made you select the particular COUNTRY you received treatment in?**<br><br>**26.1** Reputation of the Country? ☐ Yes-1 ☐ No-2

QUESTIONAIRE ON CANCER PATIENTS' CHOICE OF TREATMENT CENTER<br><br>***STUDY ID NO.______________________________***<br>***HEALTH FACILITY NAME: ________________***<br>***COUNTRY_________________________________***	
	**26.2** Your health care provider ☐Yes-1 ☐No-2<br>**26.3** Friends/ relatives ☐Yes-1 ☐ No-2<br>**26.4** Quality of care ☐Yes-1 ☐No-2<br>**26.5** Advanced medical facilities ☐Yes-1 ☐No-2<br>**26.6** Reputation for experienced health workers ☐Yes-1 ☐ No-2<br>**26.7** To combine treatment with business ☐Yes-1 ☐ No-2<br>**26.8** To combine treatment with sight-seeing ☐Yes-1 ☐No-2<br>**26.9** Cost effectiveness ☐ Yes-1 ☐No-2<br>**26.10** Other reason ☐ **26.11** Specify _____________________
	**THANK YOU VERY MUCH FOR PARTICIPATING IN THIS SURVEY.**<br>**YOUR RESPONSES WILL BE USED TO BETTER THE HEALTH SERVICES TO OTHERS**
