# Supplementary material for: Understanding and comparing the medical tourism cancer patient with the locally managed patient: A case control study
Source: PLoS One. 2022 Sep 21;17(9):e0273162. doi: 10.1371/journal.pone.0273162 (PMC9491543; doi:10.1371/journal.pone.0273162)
Supplement: S2 File — (PDF) [file pone.0273162.s002.pdf]

DODOSO LA WAGONJWA WA SARATANI NA CHAGUO LAO LA KITUO CHA MATIBABU

NAMBARI YA KITAMBULISHO CHA UTAFITI: ______________________________

JINA LA KITUO: ________________

NCHI: __________________________________

	**Maelekezo:**<br>• Tumia kalamu ya rangi na uandike wazi.<br>• Tafadhali jibu majibu yote au jaza majibu ambapo inatumika.<br>• Chapisha majibu yaliyoandikwa.<br>• Waka alama ndani ya sanduku (ikiwezekana alama ya msalaba), sio msimbo.<br>• Usiweke alama ya majibu zaidi ya moja kwa kipengee isipokua kuagizwa kufanya hivyo.<br>• Makosa yote yanafaa kusahihishwa kwa mstari mmoja.
	**Jina la mhojiwaji** ______________________________<br><br>**Je, ulikubaliana na mhojiwa?** ☐Ndio 1 ☐Hapana 2<br>Ikiwa hapana, pata kibali kabla ya kuendelea.
**1**	Tarehe ya kukamilika kwa fomu. **1.2** Nambari ya kijiunga kwa mshiriki.<br>|_|_|_|_|_|_|_|_|_| |_|_|_| dd mmm yyyy
**2**	Saa ya mahojiano **2.1** Kuanza _____ : _____ **2.2** Kumaliza: _____ :_____<br>(Saa 24)
	**SEHEMU YA B: DATA YA DEMOGRAFIA YA KIJAMII**
**3**	**Tarehe ya kuzaliwa** |_|_|_|_|_|_|_|_| *Kama hajui - 99 99 9999* **dd mm yyyy**
**4**	**Jinsia** ☐ Kiume1 ☐ Kike2
**6**	Unaishi katika **kata gani kwa sasa (Zaidi ya mwaka mmoja)?** __________________<br><br>**6.1 Makazi** yako yanapatikana wapi kwa hasa? ☐ Eneo la mijini-1 ☐ Eneo la vijijini-2
**7**	Hali yako ya **Ndoa**?<br>☐ Hajaoa 1 ☐ Katika ndoa- 2<br>☐ Wameachana- 3 ☐ Kapewa talaka- 4<br>☐ Mjane 5 ☐ Kuishi pamoja-6<br>☐ Haihusiki-7 ☐ Kakataa kujibur99

DODOSO LA WAGONJWA WA SARATANI NA CHAGUO LAO LA KITUO CHA MATIBABU

NAMBARI YA KITAMBULISHO CHA UTAFITI: ____________________________
JINA LA KITUO: _______________
NCHI:________________________________

**8**	Je! una **kiwango gani cha juu cha elimu**?
	☐ Hakuenda shule rasmi – 1 ☐ Hakukamilisha shule ya msingi - 2
	☐ Shule ya msingi – 3 ☐ Shule ya sekondari- 4
	☐ Chuo(kiwango cha kati, cheti, diploma)- 5 ☐ Chuo kikuu- 6
	☐ Shahada ya uzamili – 7
	☐ Alikataa kujibu- 99
**9**	Ni nini kinachoelezea **kazi yako kuu ?**
	☐ Mfanyakazi wa serikali - 1 ☐ Mashirika yasiyo ya serikali - 2
	☐ Hana kazi- 3 ☐ Amejiajiri- 4
	☐ Amestaafu-5 ☐ Haihusiki-6 ☐ Nyingine. 7 **9.1** Taja _____________
**10**	Umekua ukifanya kazi hii kwa **mda upi**?
	☐ Chini ya mwezi-1 ☐ Miezi 1-6-2 ☐ Miezi 7-12-3 ☐ Zaidi ya mwaka-4 ☐ Haihusiki-5
**11**	Eleza mapato yako ya wastani ya **kila mwezi katika shilingi ya Kenya? _________________**
	**SEHEMU C – MAELEZO YA UGONJWA.**
**12**	**Ni aina gani ya Saratani uliyogunduliwa nayo?**
	☐ Non Hogkins Lymphoma-1 ☐ Matumbo-2
	☐ Hodgkins Lymphoma-3 ☐ Mdomo kama- 4
	☐ Kipindi-5 ☐ Kizuizi- 6
	☐ Titi- 7 ☐ Prostate- 8
	☐ Leukemia- 9 ☐ Mfuko wa uzazi/ uterasi-10
	☐ Ubongo- 11 ☐ Utoaji -12
	☐ Mkeka wa mfupa-13 ☐ Kongosho 14
	☐ Ini- 15 ☐ Mapafu -16
	☐ Kaposi’s Sarcoma-17 ☐ Nyingine- 18 **12.1** Taja ____________

DODOSO LA WAGONJWA WA SARATANI NA CHAGUO LAO LA KITUO CHA MATIBABU

NAMBARI YA KITAMBULISHO CHA UTAFITI: ____________________________
JINA LA KITUO: _______________
NCHI:_______________________________

12.2 Ikiwa ndio, umetambuliwa Saratani kwa muda gani? |__|__|__|__|__|__|
Kama hakumbuki- 99 9999 **mm yyyy**

13 Je, umewahi ambukizwa na **magonjwa mengine ya kawaida yasiyo ya Saratani?**
□ Ndio-1 □ Hapana-2 (nenda Q 23) □ Akikataa kujibu 99 (nenda Q 23)

14

	Ugonjwa Sugu	Changia Ndio	21. Tarehe ya uchunguzi (mm/yyyy) *kama hakumbuki- 99 9999*
14.1	Shinikizo la damu		
14.2	Kisukari		
14.3	Ugonjwa wa mapafu sugu (mfano: pumu, bronchitis)		
14.4	Kunenepa zaidi		
14.5	Ugonjwa wa Hepatitis B disease		
14.6	Maradhi ya Ukimwi		
14.7	Ugonjwa wa moyo		

15 **Eleza ugonjwa wowote mwingine ambao unaweza kuwa nao? ___________**

15.1 **Kama ndio, uliambiwa una ugonjwa huu sugu lini?** |__|__|__|__|__|__|
Kama hakumbuki- 99 9999 **mm yyyy**

SEHEMU YA D: HABARI KUHUSU MATIBABU YA SARATANI

16 Je! ni **tiba gani ya Saratani** uliyopokea katika matibabu ya kwanza?
☐ Chemotherapy-1 ☐ Radiotherapy- 2
☐ Upasuaji-3 ☐ Kupandikiza mafuta ya mchanga- 4
☐ Brachytherapy – 5 ☐ Hajui-99
☐ Nyingine – 7 **16.1** Taja_____________________

17 Je! ulihitaji kutekeleza **utaratibu** wowote mwingine kwa wakati huo?
☐ PET Scan-1 ☐ Uchunguzi wa radiolojia-2
☐ Uchunguzi wa maabara-3 ☐ Hajui-99
☐ Nyingine – 4 **17.1** Taja_____________________

18 Nani au taasisi gani ililipia matibabu yako ya saratani?

☐ Fedha za kujitegemea-1 ☐ NHIF -2

DODOSO LA WAGONJWA WA SARATANI NA CHAGUO LAO LA KITUO CHA MATIBABU<br><br>*NAMBARI YA KITAMBULISHO CHA UTAFITI: ____________________________*<br>*JINA LA KITUO: _______________*<br>*NCHI:________________________________*	
	☐ Kampuni ya bima ya binafsi -3 ☐ Mwajiri -4<br>☐ Nyingine-5 **30.1** Taja ____________ ☐ Alikataa kujibu -99
**19**	**Je! wewe uliongozana na mtoaji huduma?** ☐ Ndio-1 ☐ Hapana-2 (nenda Q 22)
**20**	Ikiwa ndio, ni nani aliyesimamia gharama ya mtoaji huduma?<br>☐ Mwenyewe-1<br>☐ Wenyewe -2<br>☐ NHIF-3<br>☐ Kampuni ya bima-4<br>☐ Marafiki/ Jamaa-5<br>☐ Nyingine-6 **20.1** Taja ____________
**21**	Ikiwa ndio, uhusiano wako na mtoa huduma ni upi?<br><br>☐ Mke/Mme- 1 ☐ Rafiki- 2<br>☐ Mjumbe waamilia- 3 ☐ Nyingine – 4 **21.1** Taja____________
	**SEHEMU E. KITUO CHA MATIBABU – KWA WASHIRIKI WOTE**
**22**	**Ni kituo kipi cha afya kilikuelekeza kwa matibabu ya Saratani kwa kituo unachokwenda sasa?**<br>☐ Hospitali ya serikali -1<br>☐ Hospitali binafsi-2<br>☐ Kliniki ya kibinafsi-3<br>☐ Nyingine -4 **22.1** Taja _______________<br>**22.2** Je! Kituo kilichokuelekeza kwa kituo cha matibabu ya Saratani kiko katika kata gani?<br>______________________
**23**	**Ni nani aliyekusaidia kupanga huduma yako ya matibabu ya Saratani?**<br><br>**23.1** Mtoa huduma wako wa afya wa mtaa ☐ Ndio-1 ☐ Hapana-2<br>**23.2** Moja kwa moja na kituo unachopokea matibabu ☐Ndio-1 ☐Hapana-2<br>**23.3** Mwenyewe ☐ Ndio-1 ☐Hapana-2<br>**23.4** Marafiki na Jamaa ☐Ndio-1 ☐ Hapana-2

DODOSO LA WAGONJWA WA SARATANI NA CHAGUO LAO LA KITUO CHA MATIBABU

NAMBARI YA KITAMBULISHO CHA UTAFITI: ___________________________
JINA LA KITUO: _______________
*NCHI:*______________________________

	**23.5** Wakala wa mtaa ☐ Ndio-1 ☐ Hapana-2
	**23.6** Wakala wa ng'ambo ☐ Ndio-1 ☐ Hapana-2 ☐ Haihusiki-3
**24**	Ni **gharama** gani ya matibabu au utaratibu uliopokea?<br>(kwa dola za matibabu zinazotolewa nje ya nchi na katika shilingi ya Kenya kwa ajili ya matibabu zinazotolewa nchini Kenya.)<br>**24.1** Matibabu _______________<br>**24.2** Utaratibu _______________<br>**24.3** Malazi _______________<br>**24.4** Safari _______________
	**SEHEMU YA F : MAMBO YANAYOMSHAWISHI MGONJWA**
**25**	Ni mambo gani yaliyokufanya uamue kuchagua **KITUO CHA AFYA** ambacho umepata matibabu ya Saratani?<br>**25.1** Ukosefu wa huduma za Saratani za kutosha ambapo ulikua hapo awali?<br>☐Ndio-1 ☐ Hapana-2<br>**25.2** Kiasi cha muda ungeweza kusubiri kabla ya matibabu?<br>☐Ndio-1 ☐ Hapana-2<br>**25.3** Kama ndio, tafadhali taja muda gani ungekua umesubiri (kwa miezi) ___________<br>**25.4** Ubora bora wa huduma? ☐ Ndio-1 ☐ Hapana-2<br>**25.5** Marafiki/ jamaa? ☐ Ndio-1 ☐ Hapana-2<br>**25.6** Taarifa kutoka kwa wagonjwa wengine ambao walipata matibabu kwa eneo?<br>☐Ndio-1 ☐ Hapana-2<br>**25.7** Ushauri kutoka kwa mtoa huduma wako wa afya? ☐ Ndio-1 ☐ Hapana-2<br>**25.8** Kufuatili kambi ya matibabu nchini Kenya? ☐ Ndio-1 ☐ Hapana-2<br>**25.9** Vyanzo vya habari? ☐ Ndio-1 ☐ Hapana-2<br>**25.10** Ikiwa ndio, ni chanzo gani cha vyombo vya habari kilichoshawishi uamuzi wako?<br>☐ Mtandao-1 ☐Redio-2 ☐ Televisheni-3 ☐ Gazeti -4 ☐ Mtandao wa kijamii -5<br>☐ Nyingine-6

DODOSO LA WAGONJWA WA SARATANI NA CHAGUO LAO LA KITUO CHA MATIBABU

NAMBARI YA KITAMBULISHO CHA UTAFITI: ______________________________
JINA LA KITUO: ________________
NCHI:_________________________________

	**25.11** Eleza_________________<br><br>**25.12** Ufanisi wa gharama ya matibabu ☐ Ndio-1 ☐Hapana-2 ☐Haijihusishi-4
**26**	**Ni nini kilichokufanya uchague NCHI uliyopata matibabu?**<br><br>**26.1** Sifa ya nchi ☐ Ndio-1 ☐ Hapana-2<br>**26.2** Mtoa huduma wako ☐Ndio-1 ☐Hapana-2<br>**26.3** Marafiki/Jamaa ☐Ndio-1 ☐ Hapana-2<br>**26.4** Ubora wa huduma ☐Ndio-1 ☐Hapana-2<br>**26.5** Vifaa vya matibabu vya juu ☐Ndio-1 ☐Hapana-2<br>**26.6** Sifa kwa wafanyikazi wenye ujuzi wa afya ☐Ndio-1 ☐ Hapana-2<br>**26.7** Kuchanganya matibabu na biashara ☐Ndio-1 ☐ Hapana-2<br>**26.8** Kuchanganya matibabu na matembezi ☐Ndio-1 ☐Hapana-2<br>**26.9** Ufanisi wa gharama ☐ Ndio-1 ☐Hapana-2<br>**26.10** Sababu nyingine **26.11** Eleza _____________________
	**SEHEMU YA G: MTAZAMO JUU YA UZOEFU ULIOPATIKANA WAKATI WA MATIBABU YA SARATANI**
**27**	Je! Uzoefu wako kwa jumla umefanana na matarajio yako ya kituo cha matibabu?<br>☐ Ndio1 ☐Hapana-2
**28**	Je! Harakati yako iliwezeshwa na mtu binafsi katika taasisi? ☐Ndio-1 ☐Hapana-2<br>Ikiwa ndio, ingekua kiwango gani cha usaidizi?<br>☐Muhimu sana-1 ☐ Muhimu-2 ☐ Wastani-3 ☐Sio muhimu-4 ☐Mbaya-5
**29**	Wakati wa matibabu ulipaswa kuishi nje ya taasisi ya afya?<br>☐ Ndio1 ☐ Hapana-2

DODOSO LA WAGONJWA WA SARATANI NA CHAGUO LAO LA KITUO CHA MATIBABU

NAMBARI YA KITAMBULISHO CHA UTAFITI: ____________________________

JINA LA KITUO: _______________

NCHI:_______________________________

30 Tafadhali tupe mtazamo wako kwa mambo yafwatayo kwa kuandika jibu sahihi zaidi.

		Vizuri sana	Vizuri	Wastani	Mbaya	Mbaya sana
**30.1**	Mapokezi katika taasisi					
**30.2**	Huduma ya wateja					
**30.3**	Vituo vya makazi kwenywe taasisi au nje ya taasisi					
**30.4**	Mazingira ya taasisi ya afya					
**30.5**	Urahisi wa harakati ndani ya kituo					
**30.6**	Usafi ndani ya kituo cha afya					
**30.7**	Vifaa vya hospitalini/ taasisi					
**30.8**	Vufaa vya matibabu					
**30.9**	Vifaa vya uchunguzi					
**30.10**	Kwa heshima ya wafanyakazi wa hospitalini					
**30.13**	Ukamilifu wa huduma					
**30.14**	Kusimamia wagonjwa					
**30.15**	Utumishi wa wafanyakazi kusaidia wagonjwa					
**30.16**	Huduma ya uuguzi					
**30.17**	Msimamo wa kutunza mfanyakazi wa afya					
**30.18**	Taadhari ya kibinafsi					
**30.19**	Upatikanaji wa daktari					
**30.20**	Uwezo wa wafanyikazi wa afya kukusaidia kuelewa ugonjwa wako					
**30.21**	Uwezo wa wafanyakazi wa afya kukusaidia kuelewa matibabu/ utaratibu/ usimamizi unaohitajika					
**30.22**	Ubora wa huduma uliyopokea					

31 Je! Ungependekeza matibabu katika kituo chako cha matibabu ya Saratani kwa watu wengine?

☐Ndio$_1$ ☐Hapana$_2$ ☐ Hana uhakika$_3$

32 Ikiwa ndio, tafdhali toa sababu .

32.1 Ubora bora wa huduma nje ya nchi ☐ Ndio$_{-1}$ ☐Hapana$_{-2}$

DODOSO LA WAGONJWA WA SARATANI NA CHAGUO LAO LA KITUO CHA MATIBABU<br><br>***NAMBARI YA KITAMBULISHO CHA UTAFITI:*** ______________________________<br>***JINA LA KITUO:*** ________________<br>***NCHI:***__________________________________	
	**32.2** Ukosefu wa huduma za afya zinazohitajika nchini Kenya ☐ Ndio-1 ☐Hapana-2<br>**32.3** Upokeaji wa watumishi wa afya ☐ Ndio-1 ☐Hapana-2<br>**32.4** Ufanisi/ Gharama ya ufanisi ☐ Ndio-1 ☐ Hapana -2<br>**32.5** Ukamilifu wa huduma ☐ Ndio-1 ☐ Hapana -2<br>**32.6** Kazi ya pamoja kati ya wafanyakazi wa afya ☐ Ndio-1 ☐ Hapana -2<br>**32.7** Mengine____<br>**32.8** Eleza ____<br>____
	**ASANTE SANA KWA KUSHIRIKI KATIKA UTAFITI HUU.<br>MAJIBU YAKO YATATUMIKA KUBORESHA HUDUMA ZA AFYA KWA WENGINE.**
